# Annotations

**Published:** 1906-01-27

**Authors:** 


### ANNOTATIONS.

### Proper Schooling for the Years of Adolescence.

A CORRESPONDENT of the *Outlook* has raised this question in connection with a recent biography of Dr. Almond, of Loretto. The practice of this distinguished authority was that during the years from thirteen to seventeen physical development is the first consideration, and intellectual development a secondary one, because during these years of adolescence the calls upon the body "are so exacting that intellectual development is temporarily in abeyance." This question of athletics *versus* books is one inherently unlikely to admit of universal agreement, because those best fitted to give authoritative opinions upon the subject—to wit, schoolmasters of long experience—are certain to be biassed by their own temperamental peculiarities. For instance, the master who has attained high standing in his profession by a whole-hearted devotion to scholarship, the natural outcome of an instinctive predilection towards books, is in the nature of things likely to discount the distaste for study which in many boys is just as much the natural expression of an instinctive predilection for bodily exertion. And the same may be asserted of the contrary proposition. For ourselves, we incline, on medical grounds, to uphold the view of Dr. Almond. General experience asserts that high scholarship and a high standard of physical health are, during adolescence, mutually antipathetic, though all may be able to recall exceptions to the rule. Further, good physical health is by common consent a factor of such prime importance in human happiness that no course is justifiable which is likely to jeopardise even remotely the safety of that priceless jewel. Lastly, it is almost a platitude that excellence of scholarship is not by any means an essential in the manufacture of a successful career, while a potential taste for scholarship may very easily be annulled by ill-considered efforts to force its growth during an era which in most normal lives clamours for physical freedom.

### The Signing of Death Certificates.

AN inquiry was held on Monday evening by the Bermondsey Coroner, into the circumstances of the death of a railway guard, in consequence of an allegation by a sister of the deceased that he had been put into his coffin alive after a doctor's certificate had been given. The medical man by whom the certificate was signed, stated that the man suffered from serious heart trouble, and that on January 16 he had a stroke of apoplexy. He told a friend to prepare the wife for the worst and to let him know how the patient was at five o'clock, but at that hour a messenger came and informed him that the man had died. Having received this emphatic assurance, he considered that he was justified in giving the death certificate. Subsequently, another medical man, who made a post-mortem examination, deposed that death was due to hæmorrhage, which, judging by its extent, must have been rapidly fatal. The coroner said that the medical man in attendance on the patient made a mistake in not going to see the body before giving the certificate, and the jury, in finding that the deceased died from natural causes, added a rider to the same effect. It does not seem that in this instance any particular harm was done. But the issue of a positive death certificate on hearsay cannot in any circumstances be defended. It is true that the practice is a very common one, but this circumstance is an additional reason for its condemnation. In signing a death certificate the medical man is executing an important public duty; and even though it is performed gratuitously he should none the less appreciate the responsibility which lies upon him. Whenever the fact of death has not been personally verified, a statement to this effect should never be omitted from the certificate.

### Alcohol and Disease.

THE West Ham Guardians have decided that it is possible to treat disease without an excessive consumption of wine and spirits. Under a committee which has just handed in its report the reduction of expenditure on this item has been very marked. Thus, in the workhouse the alcohol consumed in the four weeks which ended on November 5, 1904, cost £19 19s. 1¼d., while for the corresponding period of last year the amount was only £5 6s. 0½d., although the average number of inmates was a little higher. In the infirmary the cost of alcohol during the four weeks ending November 12, 1904, was £44 2s. 11½d., the average number of patients being 602, while during the corresponding four weeks of 1905, when the average number of patients was 710, the expenditure in alcohol amounted to only £13 5s. 6½d. It is reckoned that the change in treatment will effect a reduction in the expense from £1,000 to £1,800 a year. If we thought that the economy would be injurious to the inmates of either workhouse or infirmary we should be the first to condemn it, but this is very unlikely. Indeed, it is possible that the West Ham Guardians may be able to reduce the amount of alcohol consumed still further without hurting anybody. In the Wandsworth Infirmary, where the patients are even more numerous than at West Ham, weeks pass without any alcohol being required, and when it is given it is only a matter of a few ounces for the whole institution. In short, alcohol is exclusively a medicine for emergencies. Nor do we think that the patients suffer from this limitation. On the contrary, when one considers the class who fill the beds in Poor-law infirmaries, it is safe to assume that what they want more is the knowledge that spirits of some kind or another are not necessary for the treatment of illness. It is sometimes argued that when alcohol is forbidden an equal expense may be incurred for drugs to take its place. Even if there is some truth in this contention it may be generally said that the giving of drugs, which the patient cannot easily obtain outside, is less dangerous than the resort to alcohol. In fact, the more that patients realise that alcohol is not a necessary of life either in sickness or in health, the better for them, and we believe that the moral results of the experiment will be even more beneficial than the economic.

